# Pyoderma gangrenosum presenting to an infectious diseases clinic: A 2024 case series

**DOI:** 10.1177/2050313X261463941

**Published:** 2026-07-26

**Authors:** Fabiana Kellen, Michael Silverman, Esfandiar Shojaei, MohammadReza Rahimi Shahmirzadi, Lili Ataie

**Affiliations:** 1 Schulich School of Medicine, Western University, London, ON, Canada; 2 Infectious Diseases Care Program, St. Joseph’s Hospital, London, ON, Canada

**Keywords:** pyoderma gangrenosum, PG, non-healing ulcers, post-surgical pyoderma gangrenosum, pyoderma gangrenosum diagnosis

## Abstract

Pyoderma gangrenosum is a rare neutrophilic dermatosis characterized by painful ulcerative skin lesions and a lack of definitive diagnostic tests. It is frequently mistaken for infectious conditions, often resulting in inappropriate antibiotic use and delayed immunosuppressive therapy. To describe the clinical and demographic characteristics of patients with pyoderma gangrenosum initially assessed in an infectious diseases setting and identify patterns that may aid in earlier diagnosis and management. A retrospective case series was conducted at a Cellulitis Clinic. Patients with a PARACELSUS score ⩾10 were included. Ten patients met inclusion criteria. Median age was 61.5 years; 60% were female and 90% Caucasian. Autoimmune diseases were present in 90%, mental health disorders in 50%, and endocrine abnormalities in 60%. Notably, 20% developed pyoderma gangrenosum at sites of healed scars—a rare presentation. Early recognition and multidisciplinary management are essential for timely treatment and improved outcomes.

## Introduction

Pyoderma gangrenosum (PG) is a rare and painful neutrophilic dermatosis characterized by ulcerative inflammatory skin lesions. Its clinical presentation is heterogeneous and frequently overlaps with infectious and vascular conditions, leading to frequent misdiagnosis and delays in appropriate management. PG is commonly associated with inflammatory bowel disease, inflammatory arthritis, hematologic disorders, and autoimmune conditions, with emerging evidence suggesting endocrine and metabolic associations.^1–3^ Diagnosis is challenging due to the absence of a definitive diagnostic test, and PG remains a diagnosis of exclusion supported by clinical findings and exclusion of mimickers.^4,5^

Patients with PG are often first assessed by non-dermatologists and treated for presumed infection with antibiotics before dermatologic referral, contributing to diagnostic delay and potential exacerbation through pathergy.^3,6^ This study aimed to describe the demographic characteristics, comorbidities, and clinical manifestations of PG among patients referred to an infectious disease (ID) Cellulitis Clinic, with the goal of improving recognition and reducing morbidity.

## PARACELSUS score

The PARACELSUS score is a validated diagnostic tool for PG derived from systematic analysis and control-group evaluation.^7^ The acronym reflects key diagnostic features arranged by relative frequency (Table 1).

**Table 1.** Assessment of patients with suspected PG using the PARACELSUS score.

Criteria	Points for each one present
Major criteria	
-*P*rogressive disease	3
-*A*ssessment of differential diagnosis	
-*R*eddish-violaceous wound border	
Minor criteria	
-*A*melioration by immunosuppressant drugs	
-*C*haracteristically irregular (bizarre ulcer shape)	2
-*E*xtreme pain (over 4/10 on visual analog scale)	
-*L*ocation of lesion at site of trauma	
Additional criteria	
*S*uppurative inflammation in histopathology	
*U*ndermined wound border	1
*S*ystemic disease associated	
Points ⩾10, PG is highly likely	
Points <10, PG is unlikely	

PG: pyoderma gangrenosum.

Adapted from Jockenhöfer et al.^7^

## Methods

This retrospective case series was conducted at the Cellulitis Clinic at St. Joseph’s Hospital (London, ON, USA). Between January 1, 2024 and February 28, 2025, 10 patients were diagnosed with PG based on a PARACELSUS score ⩾10.

Inclusion criteria consisted of PG diagnosis within the study window using PARACELSUS scoring. Exclusion criteria included alternative diagnoses on pathology, diagnosis outside the study window, or PARACELSUS score ⩽9.

Ethics approval was obtained from the Western University Health Sciences Research Ethics Board (#15198), with informed consent waived due to the retrospective nature of the study.

## Results

**Figure 1.**
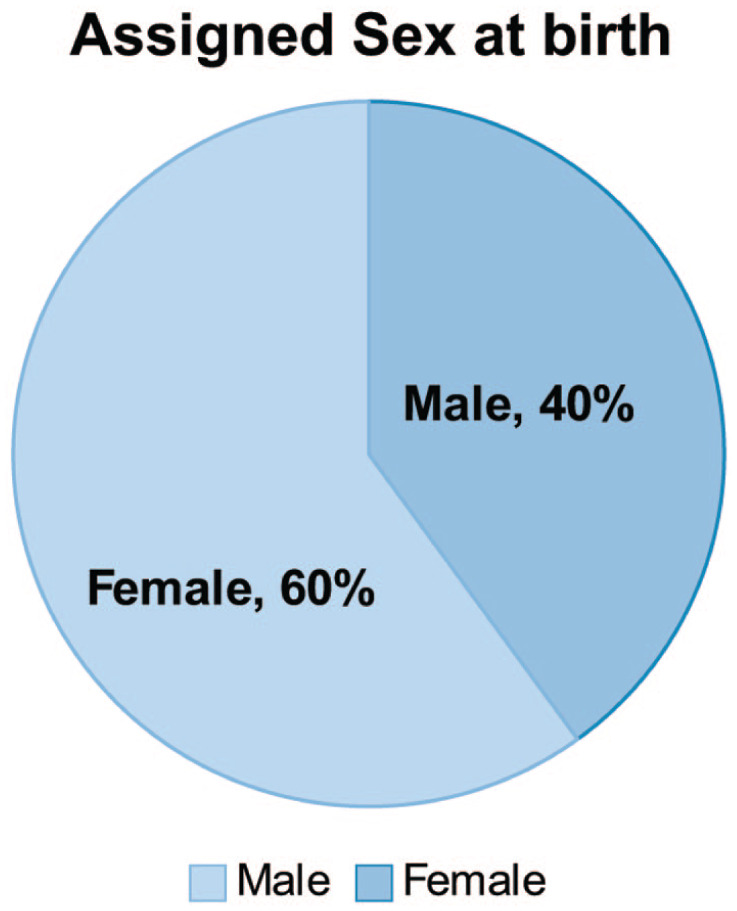
Sex distribution.

Ten patients met inclusion criteria. The median PARACELSUS score was 16 (range 10–18). Median age was 61.5 years (range 21–93); 60% were female, and 90% were Caucasian. Common comorbidities included depression or anxiety (50%), hypertension (40%), type 2 diabetes (40%), overweight (40%), obesity (30%), colitis (30%), psoriasis (30%), dyslipidemia (20%), hypothyroidism (20%), and history of malignancy (20%). None of the patients had fever at diagnosis (Figure 1). Baseline characteristics and individual patient data are summarized in Supplementary Tables 1 and 2.

**Figure 2.**
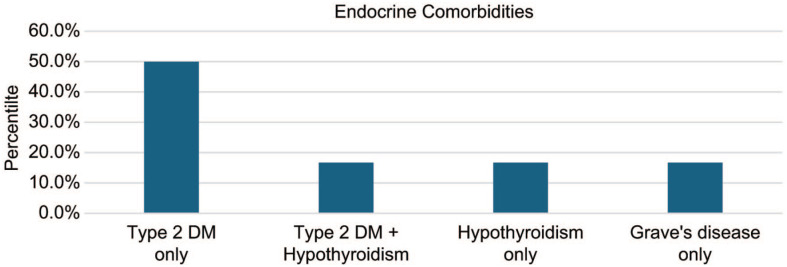
Endocrine comorbidities (*n* = 6 patients).

**Figure 3.**
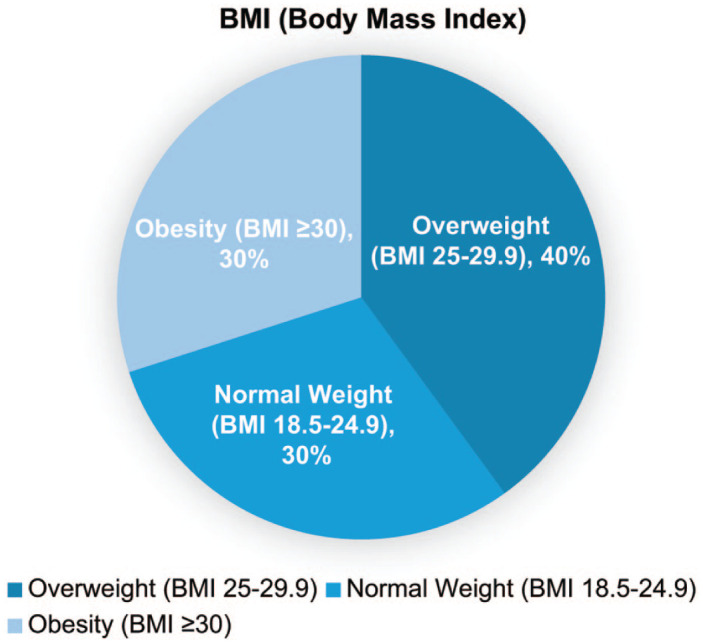
BMI distribution. BMI: body mass index.

Endocrine disorders were present in 60% of patients, including diabetes and thyroid disease (Graves’ disease and hypothyroidism). One additional patient (10%) was classified as prediabetic. Overall, 70% of patients had an elevated body mass index (BMI), suggesting a possible metabolic contribution (Figures 2 and 3).^1,2^

## Clinical presentation

**Figure 4.**
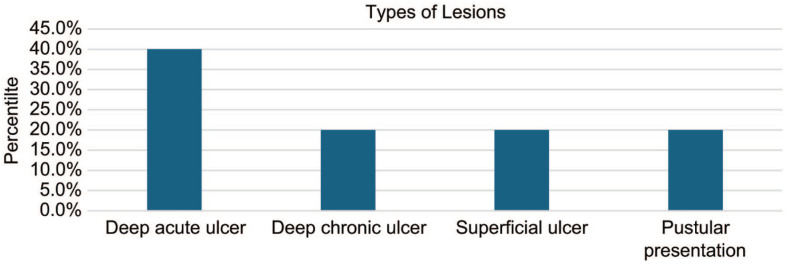
Types of lesions.

**Figure 5.**
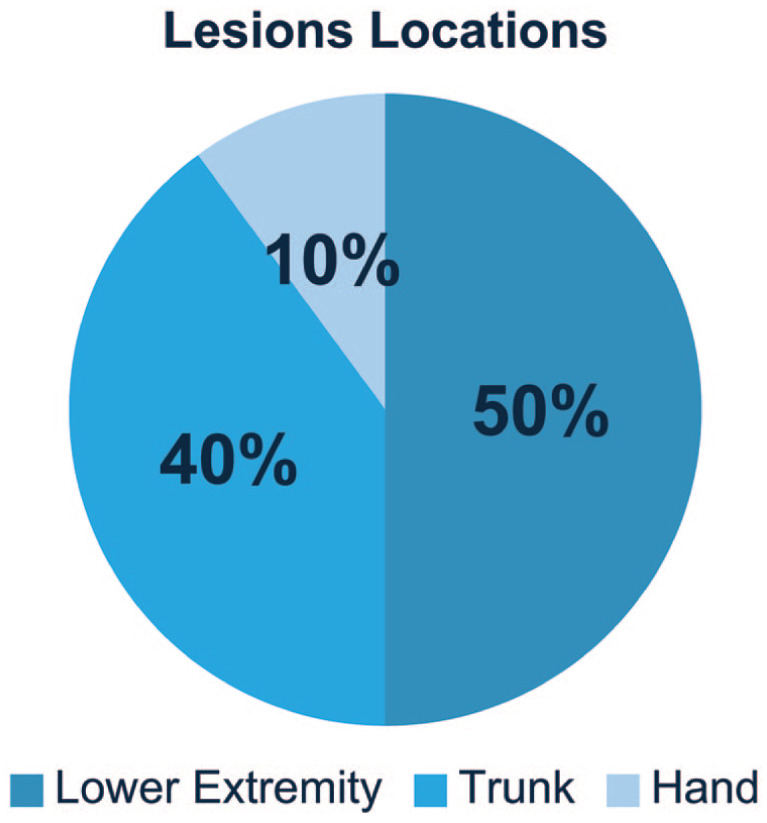
Lesions anatomical distribution.

Lesion morphology varied: 40% presented with acute deep ulcers, 20% with chronic deep ulcers, 20% with superficial ulcers, and 20% with pustular PG. No vesiculobullous lesions were observed (Figures 4 and 5).

Lesion distribution was heterogeneous. Fifty percent of lesions involved the lower legs, while remaining cases affected the abdomen, hands, buttocks, or submammary regions.

Pathergy was frequently observed. Fifty percent of patients developed PG at sites of trauma or scarring, including 20% arising in healed scars and 30% following recent injury. Undermined wound borders were present in 70% of cases.^3,6^

## Diagnostics

**Figure 6.**
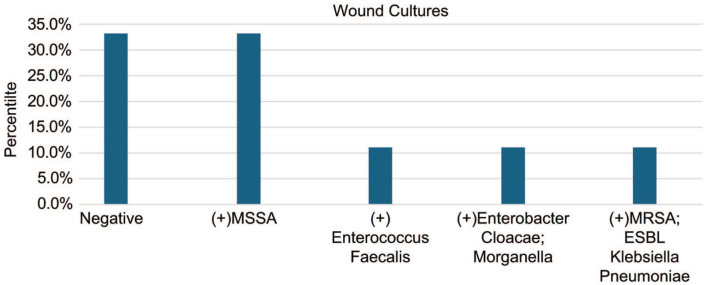
Distribution of wound culture isolates.

Histopathology was obtained in 90% of patients, but only 33% demonstrated findings supportive of PG, underscoring the limited sensitivity of biopsy and its primary role in excluding alternative diagnoses.^8,9^ Wound cultures were positive in 60%, reflecting secondary infection rather than primary etiology^10^ (Table 2 and Figure 6).

**Table 2.** Additional patients’ individual information.

Patient study ID	Typical PG findings in the biopsy	Time to Dx.*	PARACELSUS score	BMI	Cultures	Antibiotics Tx duration prior to PG diagnosis
1	Yes	5 Weeks	17	24.4	(−)	4 Weeks
2	No	23 Weeks	10	37.4	(+) *Enterococcus faecalis*	3 Weeks
3	Yes	4 Weeks	18	35.5	(−)	4 Weeks
4	Patient refused biopsy due to pain.	4 Weeks	17	27.5	(+) *Enterobacter cloacae* and *Morganella*	4 Weeks
5	Yes	40 Weeks	18	24.7	(+) MSSA	2 Weeks
6	No	3 Weeks	15	24.1	(+) MSSA	3 Weeks
7	No	4 Weeks	12	28.2	(−)	4 Weeks
8	No^a^	52 Weeks	17	28.1	(+) MSSA	3 Weeks
9	No	18 Weeks	13	35.2	(−)	16 Weeks
10	No	25 Weeks	15	28.7	(+) MRSA and ESBL *Klebsiella pneumoniae*	3 Weeks

BMI: body mass index; ESBL: extended-spectrum beta-lactamases; MRSA: methicillin-resistant *Staphylococcus aureus*; MSSA: methicillin-susceptible *Staphylococcus aureus*; PG: pyoderma gangrenosum; Tx: treatment.

* diagnosis

a Biopsy ruled out necrobiosis lipoidica and vasculitis. Some findings in favor of venous stasis.

Laboratory abnormalities were inconsistent. Leukocytosis was present in 30% and anemia in 20%, both lower than rates reported in larger cohorts.^8,11^ Serologic testing revealed anti-nuclear antibody positivity in 37.5% and perinuclear anti-neutrophil cytoplasmic antibody (p-ANCA) positivity in 12.5%. The sole p-ANCA-positive patient also had Graves’ disease.^12^ Two patients with pustular PG also had psoriatic arthritis, supporting shared immunoinflammatory mechanisms.^9,13,14^

## Treatment

All patients received multiple courses of antibiotics prior to PG diagnosis (median duration 3.5 weeks, range 2–16 weeks), reflecting frequent initial misdiagnosis.^5,15^ Following diagnosis, most patients improved with systemic corticosteroids, reinforcing the autoimmune basis of PG and the role of immunosuppression.^3,9^

## Discussion

This case series highlights the diagnostic complexity of PG in an ID setting, where lesions are frequently interpreted as primary infection. The absence of fever in all patients underscores that systemic infectious signs may be absent despite severe local inflammation.^11,15^ Histopathologic confirmation was limited, consistent with prior literature emphasizing that PG diagnosis relies on clinical judgment supported by scoring systems and multidisciplinary evaluation.^4,8^

Despite the small sample size, established PG associations were reinforced, including frequent autoimmune comorbidity and trauma-related lesion development.^3,6^ Importantly, this series also draws attention to underrecognized comorbidities. Psychiatric diagnoses were present in half of patients, exceeding previously reported prevalence and highlighting the psychosocial burden of PG.^16^ Endocrine dysfunction was common, and the high prevalence of elevated BMI suggests a potential metabolic contribution to disease susceptibility.^1,2^

Secondary infection complicates assessment in ID clinics. In this series, 60% of wounds were culture-positive, which may prompt prolonged antibiotic therapy.^10^ Recognizing PG with secondary infection is essential to avoid delayed immunosuppression and unnecessary interventions.^3^ The coexistence of pustular PG and psoriatic arthritis in two patients reinforces shared inflammatory pathways involving neutrophilic and cytokine-mediated mechanisms.^9,13^

## Limitations

The primary limitation is the small sample size inherent to rare diseases such as PG, limiting generalizability. Retrospective design and referral bias may influence findings.

## Conclusion

This study offers important insights into the clinical profile of PG within an ID context, where misdiagnosis is common. Despite a small sample size, our findings reinforce established associations—such as the frequent link to autoimmune diseases, pathergy-related lesion development, and widespread antibiotic use due to initial misidentification.

The study also draws attention to underreported yet clinically meaningful comorbidities, including a high prevalence of mental health disorders and endocrine dysfunctions like diabetes and thyroid disease. The sole patient with p-ANCA positivity also had Graves’ disease, and 70% of participants had elevated BMI, suggesting a possible metabolic contribution to disease risk.

Several key observations emerged: 60% of patients had positive wound cultures; histopathologic support was found in only 33%, highlighting diagnostic challenges. Additionally, two patients with pustular PG also had psoriatic arthritis, reinforcing immunoinflammatory overlaps.

Lesion distribution was heterogeneous: half of the patients had lower leg involvement, while the rest presented with lesions elsewhere. Notably, 20% developed PG at sites of healed scars—a rare manifestation.

Together, these findings emphasize the systemic nature of PG and highlight gaps warranting further study, particularly psychosocial and metabolic factors. Multicenter research and registry-based studies will be critical for refining diagnosis and improving long-term outcomes.

## Supplemental Material

sj-docx-1-sco-10.1177_2050313X261463941 – Supplemental material for Pyoderma gangrenosum presenting to an infectious diseases clinic: A 2024 case series Supplemental material, sj-docx-1-sco-10.1177_2050313X261463941 for Pyoderma gangrenosum presenting to an infectious diseases clinic: A 2024 case series by Fabiana Kellen, Michael Silverman, Esfandiar Shojaei, MohammadReza Rahimi Shahmirzadi and Lili Ataie in SAGE Open Medical Case Reports

sj-docx-2-sco-10.1177_2050313X261463941 – Supplemental material for Pyoderma gangrenosum presenting to an infectious diseases clinic: A 2024 case series Supplemental material, sj-docx-2-sco-10.1177_2050313X261463941 for Pyoderma gangrenosum presenting to an infectious diseases clinic: A 2024 case series by Fabiana Kellen, Michael Silverman, Esfandiar Shojaei, MohammadReza Rahimi Shahmirzadi and Lili Ataie in SAGE Open Medical Case Reports
