# Supplementary material for: Pyoderma gangrenosum presenting to an infectious diseases clinic: A 2024 case series
Source: SAGE Open Med Case Rep. 2026 Jul 26;14:2050313X261463941. doi: 10.1177/2050313X261463941 (PMC13402762; doi:10.1177/2050313X261463941)
Supplement: sj-docx-1-sco-10.1177_2050313X261463941 – Supplemental material for Pyoderma gangrenosum presenting to an infectious diseases clinic: A 2024 case series [file sj-docx-1-sco-10.1177_2050313X261463941.docx]

**Supplemental Material**

**Pyoderma Gangrenosum Presenting to an Infectious Diseases Clinic: A 2024 Case Series**

Supplementary Table 1. Characteristics of patients enrolled in our study.

| **Characteristics** | **Number (%)** |
| --- | --- |
| Median Age (age ranging from 21 to 93 y.o. ) | 61.5 y.o. |
| Female | 6 patients (60) |
| Male | 4 patients (40%) |
| Time to diagnosis in weeks median (ranging from 3 to 52 weeks) | 11.5 weeks |
| Median Paracelsus Score (Ranging from 10 to 18) | 16 |
| Fever at diagnosis |  |
| - Yes | 0 (0%) |
| - No | 10 (100%) |
| Most Common Comorbidities |  |
| - Mental health issues | 5 (50%) |
| - Hypertension | 4 (40%) |
| - Type 2 Diabetes | 4 (40%) |
| - Colitis | 3 (30%) |
| - Dyslipidemia | 3 (30%) |
| - Psoriasis | 3 (30%) |
| - Obesity | 3 (30%) |
| - Overweight | 4 (40%) |
| - Hypothyroidism | 2 (20%) |
| - Hx of cancer (one pt had treated breast cancer and the other had treated renal cell carcinoma) | 2 (20%) |
| Leukocytosis at diagnosis | n=10 patients (100%) |
| - Normal levels | 7 (70%) |
| - Elevated levels (ranging from 10.4x10^9^/L to 13.8x10^9^/L; median: 11.7x10^9^/L) | 3 (30%) |
| Neutrophilia at diagnosis | n=10 patients (100%) |
| - Normal levels | 8 (80%) |
| - Elevated levels (ranging from 8.2x10^9^/L to 11.4x10^9^/L; average: 9.8x10^9^/L) | 2 (20%) |
| Hemoglobin at diagnosis | n=9 patients (90%) |
| - Normal levels | 7 (78%) |
| - Decreased levels (ranging from 116g/L to 129g/L; average: 122.5g/L) - Normal MCV (91.3fL) - Elevated MCV (103.3fL) | 2 (22%)  1 (50%)  1 (50%) |
| Hb1Ac | n=10 patients (100%) |
| - Normal levels | 6 (60%) |
| - Elevated levels (ranging from 6.2% - 8.8%; median: 6.9%) | 4 (60%) |
| CRP | n=10 patients (100%) |
| - Normal levels | 3 (30%) |
| - Elevated levels (ranging from 5.1mg/L – 33.2mg/L; median: 17.4mg/L) | 7 (70%) |
| Creatine | n=10 (100%) |
| - Normal levels | 9 (90%) |
| - Elevated levels (132 µmol/L) | 1 (10%) |
| Immunology | n=* |
| - p-ANCA normal levels | 7 (88.4%) |
| p-ANCA elevated levels (58RU/ml) | 1 (12.5%) |
| - c-ANCA normal levels | 8 (100%) |
| - ANA negative | 5 (62.5%) |
| ANA positive (ranging from 1/80 to 1/160; median: 1/80) | 3 (37.5%) |
| - RF negative | 3 (60%) |
| RF positive (ranging from 15 IU/ml to 18 IU/ml; median: 16.5 IU/ml) | 2 (40%) |
| - Alpha 1 Globulin normal levels | 5 (83%) |
| Alpha 1 Globulin elevated levels (5.1g/L) | 1 (17%) |
| - Alpha 2 Globulin normal levels | 3 (50%) |
| Alpha 2 Globulin elevated levels (ranging from 9.9g/L to 10.5g/L; median: 10.3g/L) | 3 (50%) |
| - Beta 1 and Beta 2 Globulin normal levels | 5 (83%) |
| Beta 1 and Beta 2 Globulin elevated levels (6.3g/L) | 1 (17%) |
| - Gamma Globulin normal levels | 5 (83%) |
| Gamma Globulin decreased levels | 1 (17%) |
| - Light Chains Kappa normal levels | 6 (75%) |
| Light Chains Kappa increased levels | 2 (25%) |
| - Light Chains Lambda normal levels | 7 (87.5%) |
| Light Chains Lambda increased levels | 1 (12.5%) |
| - Light Chains Kappa/Lambda ratio normal levels | 8 (100%) |
| - Hepatitis B and C | n=7 patients (70%) |
| Positive | 0 (0%) |
| - Negative | 7 (100%) |
| Wound Biopsy | n=9 patients (90%) |
| - Positive typical findings for PG | 3 (33%) |
| - Negative typical findings for PG | 6 (67%) |
| Wound Culture: number of positive cultures | n=10 patients (100%) |
| - Negative | 4 (40%) |
| - Positive | 6 (60%) |
| Wound Characteristics | n=10 patients (100%) |
| - Deep acute ulcer | 4 (40%) |
| - Deep chronic ulcer | 2 (20%) |
| - Superficial ulcer | 2 (20%) |
| - Pustular presentation | 2 (20%) |
| - Vesiculobullous lesion | 0 (0%) |
| Undermined Wound Margin | n=10 patients (100%) |
| - Yes | 7 (70%) |
| - No | 3 (30%) |
| Wound location | n=10 patients (100%) |
| - Lower leg | 5 (50%) |
| - Abdomen | 2 (20%) |
| - Buttocks | 1 (10%) |
| - Hands | 1 (10%) |
| - Submmamary | 1 (10%) |
| Treatment: Antibiotics received prior to PG diagnosis | n=10 patients (100%) |
| - Duration of antibiotics treatment mean (ranging from 2-16 weeks) | 3.5 weeks |

** Alpha 1 and 2 Globulin, Beta 1 and 2 Globulin, and Gamma Globulin tests, n=6.; ANA (Anti-Nuclear Antibody), c-ANCA (cytoplasmatic Anti-Neutrophil Cytoplasmatic Antibody), and p-ANCA (perinuclear Anti-Neutrophil Cytoplasmic Antibody) tests, n=8; Light Chanis Kappa, Lambda, and k/l ratio tests, n=8. RF (Rheumatoid Factor), n=5;*
