# Supplementary material for: Pyoderma gangrenosum presenting to an infectious diseases clinic: A 2024 case series
Source: SAGE Open Med Case Rep. 2026 Jul 26;14:2050313X261463941. doi: 10.1177/2050313X261463941 (PMC13402762; doi:10.1177/2050313X261463941)
Supplement: sj-docx-2-sco-10.1177_2050313X261463941 – Supplemental material for Pyoderma gangrenosum presenting to an infectious diseases clinic: A 2024 case series [file sj-docx-2-sco-10.1177_2050313X261463941.docx]

**Supplemental Material**

**Pyoderma Gangrenosum Presenting to an Infectious Diseases Clinic: A 2024 Case Series**

Supplementary Table 2. Patients’ individual information.

| **Patient Study ID** | **Demographics;**  **Wound location and Characteristics*** | **Comorbidities** | **History of Presenting Illness** | **Lesion Description** |
| --- | --- | --- | --- | --- |
| 1 | 73 y.o. F  From left groin to lower abdominal area (1)^*^ | -Grave’s disease  -Grave’s ophthalmopathy  -elevated pANCA (58 RU/mL)  -Psoriasis | Patient reported that she cut herself while trimming her pubic hair. The lesion increased in size and did not improve despite over 3 weeks of IV antibiotics. It responded to oral corticosteroids.  p-ANCA was elevated at 58. | Large ulcer with red-violaceous border which was friable and bled easily. |
| 2 | 58 y.o.  Left lower leg; (3)* | -Hypertension  -Hypothyroidism  -Obesity  -PUD  -Hyperglycemia (steroid induced)  -Type 2 diabetes  -Psoriatic arthritis on Secukinumab  -Psoriasis | Patient reported painful lesions on legs and abdomen with increased intermittent swelling around the ankles. Four months later, there was a superficial ulceration on left lower leg above medial malleolar area. Patient had moderate improvements over eighteen months after several courses of antibiotics and oral corticosteroids. | A large patch of erosive pustular ulcer on the lower extremity. |
| 3 | 62 y.o. M  Left middle finger and right dorsal hand (1)* | -GERD  -Mental health issues (depression)  -Type 2 diabetes  -Obesity  -Esophageal spasm  -Dyslipidemia  -Remote hx of Rheumatic fever  -Hx of focal active colitis (confirmed on biopsy as a non-IBD type) | Recurrent dorsal hand and finger ulcers since 2018 which were initially treated as skin infections. In May, 2024 a biopsy confirmed pyoderma gangrenosum, and his condition quickly responded to oral and topical corticosteroids. However, he had recurrent lesions every few months. | - Right dorsal hand had two ulcers with slight discharge and purplish margins.  -Ulcer on left middle finger with violaceous border and tenderness. |
| 4 | 68 y.o. F  Left medial leg (1)* | -Hypertension  -Seasonal allergies  -Mental health issues (depression)  -Hx of breast cancer (mastectomy)  -Essential tremors  -Chronic intermittent diarrhea/ microscopic colitis (colonoscopy ruled out IBD)  -Overweight | Patient had a scar on her left leg due to a fall that occurred many years ago. This scar began to swell and cause pain with fast ulceration. She was treated with several courses of antibiotics and incision and drainage at the emergency department. Non-responsive to IV and oral antibiotics but with fast response to topical potent steroid. Her ANA was 1/80 and her RF was slightly elevated at 18 IU/ml. | Painful ulcer with purple undermining edge. Ulcer with no significant discharge; periwound erythema and tenderness present. |
| 5 | 93 y.o. M  Abdomen (4)* | -Ulcerative colitis  -Type 2 diabetes  -Diabetic retinopathy  -Overweight  -Chronic kidney disease  -COPD  -Hypertension  -Dyslipidemia  -Gout  -Transient Ischemic Attack  -Heart failure with preserved ejection fraction  -Type 2 AV block  -Peripheral vascular disease with occluded femoral artery and distal popliteal artery  -Hx of recurrent pneumonia  -Hx of stage T1A renal cell carcinoma with left partial nephrectomy | Almost one year prior to presentation at the ID clinic, patient noticed a growing ulcer at the site of a previous laparoscopic nephrectomy. Antibiotics prescribed prior to his assessment at the ID clinic were reportedly not helpful. The ulcer responded to oral and topical corticosteroids and it was healed after two months of treatment. | Large superficial moist ulcer with discharge and mucoid layer over it. There was a small satellite dry lesion located caudally to the primary ulcer. There was no pus, and the wound was not surrounded by cellulitis.  The primary lesion was present for about 10 months, and it had increased and decreased its size overtime. |
| 6 | 65 y.o. F  Right medial leg (1)* | -Chronic prurigo  -Mental health issues (anxiety and depression)  -Dyslipidemia  -Hypertension  -Hypothyroidism  - History of acne during adolescence | Hx of chronic prurigo lasting for 1.5 years prior to presentation at the ID clinic. Patient developed lesions from scratching her skin (upper and lower extremities). Topical steroids and antihistamines helped to control the prurigo slightly. An ulcer developed on one of the excoriation areas. The ulcer responded to oral and topical corticosteroids, and it was cleared in two months. ANA was 1/160. | Large open ulcer which was oozing and tender. There was surrounding erythema and the lesion had a “heart shape”. |
| 7 | 27 y.o. F  Right anteromedial mid shin (3)* | -Mental health issues (depression and other disorders)  -Eating disorder  -Overweight  -Irritable bowel syndrome  -Alcoholic fat liver disease  -Psoriasis  -Psoriatic arthritis not yet on immunomodulators  -Sebopsoriasis of face | Patient had underlying psoriasis and was referred to the ID clinic for inflammatory pustular swelling on right leg for the past four weeks. It had not improved on several courses of antibiotics, and was getting more inflamed and erythematous. It responded moderately to a two-week course of potent topical corticosteroids. RF was slightly elevated at 15 IU/ml. | Tender swelling on right leg with superimposed pustular lesions and peripheral erythema. |
| 8 | 37 y.o. M  Two ulcers on left lower leg (shin). (2)* | -Type 2 diabetes (insulin dependent)  -Overweight  -Mental health disorders (anxiety and depression) | The patient reported a left leg injury one year ago, which subsequently developed into an ulcer that progressively worsened. The ulcer did not improve with oral antibiotics and continued to deteriorate. A secondary lesion later developed distal to the original site. After being referred to the ID clinic there was notable improvement with one week of potent topical corticosteroid and significant reduction in ulcer size by five weeks. | Two ulcers surrounded by purplish erythema with some serosanguineous discharge. Moderately painful. |
| 9 | 21 y.o. F  Right submammary area (4)* | -Hidradenitis suppurativa  -Hx of keloids  -Hx of acne  -Obesity  -Borderline type 2 diabetes | Patient reported a chronic ulcer that started as a small boil and gradually increased in size. Prior to ID clinic referral, patient was treated with several courses of antibiotics over a period of four months with no improvements. Fungal and mycobacterial tissue cultures were negative. Patient responded moderately to an intralesional steroid injection. | Superficial ulcer with significant serous discharge without purplish rim. |
| 10 | 61 y.o. M  Left buttocks (2)* | -Chronic pain (query fibromyalgia)  -Pancreatitis  -Overweight | The patient reported that the ulcer began as a pimple approximately five months ago and gradually increased in size. Patient denied being bedridden. In addition, patient did not have signs of peripheral neuropathy or impaired sensation in the saddle area. Prior to ID clinic referral, patient received several courses of antibiotic treatment with no significant improvement. Marked improvement was observed after four weeks of treatment with potent topical corticosteroids. ANA was 1/80. | Well circumscribed ulcer with a clean base and purplish rim. |

*F = Female; M= Male; IBD: Irritable Bowel Disease; PUD: Peptic Ulcer Disease*

*^*^Wound Characteristics: (1) Acute deep ulcer, (2) Chronic deep ulcer, (3) Pustular presentation, (4) Superficial ulcer, (5) Vesiculobullous lesion*
